# Rating versus ranking in a Delphi survey: a randomized controlled trial

**DOI:** 10.1186/s13063-023-07442-6

**Published:** 2023-08-18

**Authors:** Claudio Del Grande, Janusz Kaczorowski

**Affiliations:** 1 Health Innovation and Evaluation Hub, University of Montreal Hospital Research Centre, Saint-Antoine Tower, 850 Saint-Denis Street, Montreal, QC H2X 0A9 Canada; 2 School of Public Health, Université de Montréal, Montreal, QC Canada; 3 Department of Family Medicine and Emergency Medicine, Université de Montréal, Montreal, QC Canada

**Keywords:** Delphi technique, Randomized controlled trial, Health services research, Primary health care, Rating, Ranking, Reliability

## Abstract

**Background:**

The Delphi technique has steeply grown in popularity in health research as a structured approach to group communication process. Rating and ranking are two different procedures commonly used to quantify participants’ opinions in Delphi surveys. We explored the influence of using a rating or ranking approach on item prioritization (main outcome), questionnaire completion time, and evaluation of task difficulty in a Delphi survey aimed at identifying priorities for the organization of primary cardiovascular care.

**Methods:**

A randomized controlled parallel group trial was embedded in a three-round online Delphi survey. After an “open” first round, primary care patients, trained patient partners, and primary care clinicians from seven primary care practices were allocated 1:1 to a rating or ranking assessment group for the remainder of the study by stratified permuted block randomization, with strata based on participants’ gender and status. Agreement on item prioritization between the experimental groups was measured by calculating Krippendorff’s alpha reliability coefficient on the aggregate rank order of items in each group after the final round. Self-reported ease or difficulty with the assessment task was measured with the Single Ease Question.

**Results:**

Thirty-six panelists (13 clinic patients, 7 patient partners, 16 clinicians; 60% females) were randomized to the rating (*n* = 18) or ranking (*n* = 18) group, with 30 (83%) completing all rounds. Both groups identified the same highest priorities from a set of 41 items, but significant discrepancies were found as early as the seventh top item. There was moderately strong agreement between the priority ordering of top items common to both groups (Krippendorff’s alpha = 0.811, 95% CI = 0.669–0.920). A 9-min mean difference to complete the third-round questionnaire in favor of the rating group failed to achieve statistical significance (*p* = 0.053). Ranking was perceived as more difficult (*p* < 0.001).

**Conclusions:**

A rating or ranking procedure led to modestly similar item prioritization in a Delphi survey, but ranking was more difficult. This study should be replicated with a larger number of participants and with variations in the ranking and rating procedures.

**Trial registration:**

Not applicable.

**Supplementary Information:**

The online version contains supplementary material available at 10.1186/s13063-023-07442-6.

## Background

The Delphi technique is a structured approach to group communication process developed by the RAND Corporation in the 1950s to obtain more reliable opinion from knowledgeable individuals [1]. Initially applied to forecasting, the technique quickly proved to be relevant in many other areas to support decision-making in contexts of uncertainty, when one must rely, at least in part, on subjective judgment of experts in the absence of definitive evidence [2–4]. Over the past decades, Delphi has steeply grown in popularity in health research, where it has been used for various purposes such as the selection of core outcome sets, quality indicators, or research priorities. The Delphi technique is based on four key components—anonymity, iteration, controlled and systematic feedback, and statistical aggregation of group response—favoring indirect communication through rounds of questionnaires rather than direct confrontation of participants [4, 5]. By doing so, Delphi avoids common deficiencies associated with in-person meetings (e.g., undue influence of personal characteristics and social factors, time limits, subjective summaries) and effectively removes geographic and time boundaries, allowing dispersed individuals to asynchronously partake and contribute to the communication process democratically. Incidentally, this also makes the technique ideally suited to settings where contact reduction measures, such as those implemented during the recent COVID-19 pandemic, are in effect.

Despite the “fixed” components that are its hallmark, Delphi studies show great variability in their implementation. Over the years, many reviewers have criticized and attempted to overcome the lack of guidance compromising the validity of Delphi outputs [6–16]. Recently, a series of empirical studies examined various methodological aspects of the technique more closely. Brookes et al. [17] reported that providing feedback from all stakeholder groups separately in heterogenous panels may influence the items retained and improve consensus compared to providing peer group feedback only. However, MacLennan et al. [18] found no such effect in a context of high initial agreement over the items appraised. Brookes et al. [19] also noted that question order may influence the items ultimately retained. Gargon et al. [20] discovered that larger panels and larger item pools were independently associated with lower response rates. De Meyer’s [21] and Lange’s [22] teams both observed that varying the number of response categories on the rating scale and the consensus criteria led to widely different outcomes. Finally, Boel et al. [23] reported that inviting panelists to subsequent rounds regardless of their response status in the previous round improved response rate without significantly changing the Delphi outcome. Such studies are important in allowing end-users to consider Delphi results more thoughtfully and future investigators to support their study design choices with evidence.

However, the choice of assessment procedure to quantify the opinion of panel members is a key methodological aspect of the Delphi technique that has remained unexplored to date. In this regard, rating and ranking are the most commonly used [4, 24]. The conduct of rating and ranking Delphi studies is very similar, but the latter may include an intermediate step between item elicitation and item assessment for narrowing down the list of items to a manageable number for ranking (20 or less, as suggested by Schmidt [25]). There is no simple mathematical solution to interchange rankings and ratings [26]. Each assessment mode has advantages and disadvantages over the other. Ranking provides a relative order of appreciation and forces the prioritization of items. It prompts thoughtful consideration of all items in the list, which can enhance response quality. However, this comes at the price of an increasing cognitive burden on respondents as the list increases in size [27–29]. Moreover, rankings yield no information about the gap between consecutively ranked items. Since a potentially large or insignificant difference cannot be accounted for, ranking Delphi investigators may unknowingly choose suboptimal cutoffs for the inclusion or exclusion of items upon study completion. Conversely, rating provides an absolute order of magnitude as to how far apart two items are in terms of appreciation along a given scale. However, requiring less effort and allowing ties, rating is susceptible to agreement bias—a tendency to respond positively regardless of the content of the item—and non-differentiation, leading to less variation in scores across items [27–29]. This is more likely to occur when most items have a desirable connotation, a situation that is common in health Delphi studies. This often results in large proportions of items being retained (e.g., [18, 30, 31]), a situation which poses a greater challenge for knowledge mobilization in real-world settings.

Most of the empirical literature to date comparing rating and ranking is in the field of value systems measurement in psychology [32]. The evidence shows that the two procedures produce similar results in the aggregate and may be interchangeable for the purpose of measuring average preference orderings [27–29, 33]. At the individual level, rating and ranking would seem to allow respondents to discriminate equally well between extreme items (most/least favored), but not between items of moderate importance where rating tends to underperform compared to ranking [34]. However, value systems have been shown to exhibit underlying structural properties (e.g., intrinsic or extrinsic) persisting across assessment modes, which may bolster the comparability of rating and ranking [35]. Thus, whether these findings apply in contexts where items do not present such latent structures, as is typically the case in Delphi studies, remains unknown. The main objective of this study was to determine whether a rating or ranking approach would lead to different item prioritization in a Delphi study. The secondary objectives were to determine the effect of assessment mode on questionnaire completion times and ease of panelists with the assessment task.

## Methods

### Study design and population

A randomized controlled parallel group trial design embedded in an online Delphi study was used to conduct this research. The Delphi study aimed to identify the top organizational priorities shared by patients and clinicians regarding the management of cardiovascular diseases and risk factors in primary care settings. The main results of the study have been published elsewhere [36]. This paper focuses on the methodological comparison between rating and ranking.

Participants were regular primary care patients, formally trained patient partners experienced in the mobilization of their experiential knowledge, and primary care clinicians. Regular patients and clinicians were recruited opportunistically from seven family medicine group settings operating in metropolitan, suburban, and remote areas in the province of Quebec (Canada), by using posters in waiting rooms and staff rooms, and flyers distributed during on-site visits and lunch conferences. Patient partners rostered in Université de Montréal’s Patient Collaboration and Partnership branch [37] were invited by email by the branch coordinator. To be eligible, patients had to be over 18 years old and diagnosed with established cardiovascular disease or a clinical cardiovascular risk factor such as hypertension or dyslipidemia. Eligible clinicians included family physicians, nurses and other allied healthcare professionals providing cardiovascular care to patients.

#### Sample size

This nested trial was opportunistic in nature and statistical hypothesis testing should be viewed as exploratory. The sample size was determined by the number of participants in the Delphi study, which aimed to recruit 40 participants roughly balanced between patients and clinicians. Assuming a 15% loss to follow-up, this sample size allowed for two subpanels of 17 participants each (the rating and ranking study groups), which was equal to the median number of panelists found in a systematic review of Delphi studies published in healthcare [9]. Unlike traditional surveys, larger samples are not necessarily preferred in Delphi surveys because they aim to refine participants’ opinions over the course of iterative rounds, rather than provide a cross-sectional representation of them. This process is known to become unwieldy and suboptimal as the number of participants increases, especially when reasons are fed back to participants along with statistical summaries, which was the case in the present study [15, 38, 39].

#### Ethical considerations

The study was performed in accordance with the ethical standards of the Declaration of Helsinki [40] and with ethical approval by the University of Montreal Hospital Research Centre’s research ethics committee (project number 17.305). This trial did not require registration because neither the assigned interventions nor the outcomes assessed were related to the health of participants.

### e-Delphi process

The Delphi process took place entirely online on the SurveyMonkey platform. The number of rounds was predetermined at three to minimize attrition and because this was deemed sufficient to identify the prioritization of items [9, 41]. Participant recruitment and data collection occurred over a 1-year period, from November 2019 to November 2020. All study questionnaires were pretested with nonparticipating patients and clinicians (*n* = 4). Up to three reminders were sent to nonrespondents two weeks apart during each round. The first round was “open” and conducted prior to randomization, to ensure that both study groups would have the same item pool. During this round, each panelist submitted up to five important organizational items for primary cardiovascular care in free-text and answered sociodemographic and general health questions. Items were then synthesized and grouped into thematic lists to facilitate their initial assessment.

#### Randomization

After round 1, participants were randomly allocated 1:1 to a rating or ranking assessment procedure for the remainder of the Delphi process, which proceeded independently in the study groups. Stratified permuted block randomization was performed without involvement of the researchers by the CHUM Center for the Integration and Analysis of Medical Data (CITADEL), with strata based on panelists’ gender (female, male) and status (clinic patient, faculty patient partner, clinician) to balance the study groups. Participants’ anonymity was maintained throughout the study and panelists were unaware of the experiment. While it was not possible to blind participants to their assigned assessment procedures because of the nature of the interventions, they were kept unaware of the study hypotheses, and there were no interactions between participants allocated to different groups or with the study team. The assessors who evaluated the outcomes were aware of the participant allocation, but they could not influence the classification of panelists’ responses. All quantitative assessments and analyses were conducted without requiring subjective interpretation.

#### Rating and ranking procedures

During the second and third rounds, panelists were sent the questionnaire version that made use of their assigned assessment mode to appraise the items. Aside from specific instructions regarding each assessment procedure and related survey design questions, the content of both versions of the survey questionnaires was identical. In the second-round questionnaires, items were assessed within their thematic list on separate pages. To prevent order effects, the lists and items within each list were presented in random order to each panelist. Rating panelists used a 7-point unipolar scale ranging from 1 “not at all” to 7 “extremely” important. Numeric and verbal labels were added for each response category to reduce measurement error and increase reliability and validity [15, 42, 43]. Ranking panelists placed the items in each thematic list in descending order of importance (top = most important). A narrowing-down step was not required in the ranking group because the largest list contained only seven items [11, 25]. Panelists were encouraged to provide reasons in free-text fields to support their assessments as an essential means to circulate valuable knowledge among them [15, 44].

During the final round, panelists received structured statistical and qualitative feedback from their study group including, for each item: the person’s score, the group’s median rating or ranking, the interquartile range (IQR), and a summary of positive and negative reasons formulated. They were asked to reappraise only a subset of them to keep their focus on potential top priorities. This subset was produced independently in the two study arms. In the rating group, it included items scored 6 or 7 by at least two-thirds of panelists. In the ranking group, it included items ranked in the top half of their list by at least two-thirds of panelists. These criteria seemed sufficiently comparable as the items had been freely elicited as important from the outset, and we anticipated that most ratings would lie between 4 and 7. This time around, the items were presented on a single list in descending order of importance, as recommended by Delphi methodologists [11, 25]. To reduce the cognitive burden put on ranking panelists, they were asked to select their top-half among the subset of items before proceeding to ranking their top and bottom halves separately. Full ranking was reconstructed at the analysis stage. Final priorities in each study group were determined by rank-ordering the items in their subset based on the proportion of 6–7 ratings obtained in the rating arm and of top-half rankings in the ranking arm. To break ties, 7-only ratings and median ranks were used in the rating and ranking groups, respectively.

### Trial outcomes and statistical analyses

The main outcome of this trial was the level of agreement on the top organizational items between the two study groups after round 3. This was assessed by examining the overlap between the most important items in each group and by calculating Krippendorff’s alpha coefficient on the aggregate rank order of items in the two groups. Krippendorff’s alpha is a reliability measure that has been proposed as the standard reliability statistic due to its flexibility and advantages over other known reliability coefficients [45, 46]. The coefficient can take any value between − 1 (inverse agreement or perfect systematic disagreement) and 1 (perfect agreement), with 0 indicating absence of agreement beyond chance. Bootstrap (*n* = 10,000 samples) was used to produce 95% confidence intervals.

The secondary outcomes were as follow: (1) time to complete round two and round three questionnaires, measured separately, and (2) self-reported ease or difficulty with the assessment task, measured by the Single Ease Question (SEQ) administered immediately after the final assessment of items. The SEQ is a standard user metric in the form of a 7-point rating scale (from 1 “very difficult” to 7 “very easy”) that is simple, quick, and has proven to perform as well or better than more complicated measures of task difficulty [47]. The study questionnaires were designed to take around 20 min to complete. Assuming a standard deviation of 7 min, the minimum difference between the study groups that would be detectable with a sample size of 34, with *α* = 0.05 and 80% power, is 7.8 min. The average SEQ score has been estimated to be around 5.5 across over 400 tasks and 10,000 users [47]. Assuming a standard deviation of 1, the minimum detectable difference under the conditions of this trial was 1.1. Secondary outcome measures were compared between the study groups using Mann–Whitney *U* tests, due to the ordinal nature of the SEQ and violations found in the normality assumption of the questionnaire completion time data, making *t*-tests inappropriate. Fisher’s exact tests were used to compare the characteristics of panelists in the two experimental groups due to the small sample sizes. Statistical analyses were performed in SPSS version 26 (IBM Corp.). Statistical significance level was set at *p* < 0.05.

## Results

**Fig. 1:**
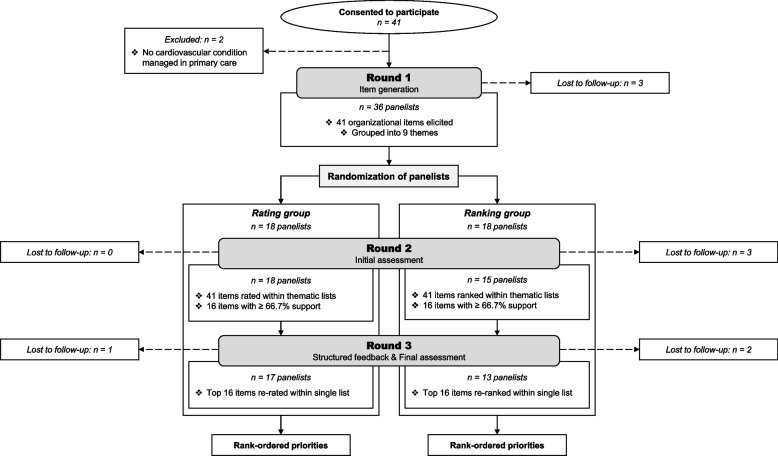
Flow chart of the study

Figure 1 depicts the study flow. A total of 41 individuals consented to participate. However, two were found to be ineligible and three never accessed the first questionnaire after giving consent. Thus, 36 panelists (20 patients and 16 clinicians: 10 family physicians, 5 registered nurses, and 1 allied healthcare professional) were randomized, with 30 of them (83%) completing all rounds. Attrition was evenly distributed over the Delphi rounds but mostly occurred in the ranking group (5 dropouts vs. 1 in the rating group; Fisher’s exact *p* = 0.177).

Table 1 presents the characteristics of Delphi panelists. The profile of participants remained balanced between the experimental groups throughout the Delphi process, with no statistically significant differences found on main covariates.

**Table 1:** Profile of Delphi panelists

	**First round, *n* (%**^a^**)**			**Third round, *n* (%**^a^**)**		
	Rating group (*n* = 18)	Ranking group (*n* = 18)	*p*^†^	Rating group (*n* = 17)	Ranking group (*n* = 13)	*p*^†^
**Participant category**			1.000			0.889
Regular clinic patients	6 (33.3)	7 (38.9)		6 (35.3)	3 (23.1)	
Faculty patient partners	4 (22.2)	3 (16.7)		4 (23.5)	3 (23.1)	
Clinicians	8 (44.4)	8 (44.4)		7 (41.2)	7 (53.8)	
**Gender**			1.000			0.440
Female	10 (58.8)	11 (61.1)		10 (58.8)	10 (76.9)	
Male	7 (41.2)	7 (38.9)		7 (41.2)	3 (23.1)	
**Age group**			0.907			1.000
18–34	6 (35.3)	5 (27.8)		6 (35.3)	5 (38.5)	
35–49	4 (23.5)	3 (16.7)		4 (23.5)	2 (15.4)	
50–64	4 (23.5)	4 (22.2)		4 (23.5)	3 (23.1)	
65–79	2 (11.8)	4 (22.2)		2 (11.8)	2 (15.4)	
80 +	1 (5.9)	2 (11.1)		1 (5.9)	1 (7.7)	
**Education level**			0.687			0.720
High school	2 (11.8)	0 (0)		2 (11.8)	0 (0)	
College/vocational	4 (23.5)	5 (27.8)		4 (23.5)	2 (15.4)	
University	10 (58.8)	12 (66.7)		10 (58.8)	10 (76.9)	
Preferred not to answer	1 (5.9)	1 (5.6)		1 (5.9)	1 (7.7)	
**Perceived general health**			0.690			0.871
Excellent or very good	7 (41.2)	10 (55.6)		7 (41.2)	7 (53.8)	
Good	8 (47.1)	7 (38.9)		8 (47.1)	5 (38.5)	
Fair or poor	2 (11.8)	1 (5.6)		2 (11.8)	1 (7.7)	
**Setting**			0.480			0.895
Metropolitan	7 (38.9)	10 (55.6)		6 (35.3)	6 (46.2)	
Suburban	6 (33.3)	3 (16.7)		6 (35.3)	3 (23.1)	
Remote	5 (27.8)	5 (27.8)		5 (29.4)	4 (30.8)	

^†^Fisher’s Exact tests (two-tailed exact significance)

^a^Valid percentages, not accounting for missing values

Forty-one mutually exclusive organizational items were elicited during round 1. These were grouped under nine themes: accessibility (7 items), services network (5 items), care and follow-up (4 items), self-management support (4 items), clinical team composition (6 items), professional collaboration (5 items), professional training (3 items), patient-professional relationship (3 items), and information systems (4 items). All results of the initial assessment of items during Round 2 in each study group are provided in Additional file 1.

### Agreement on top priorities

The subset of items reassessed in the final round coincidentally included 16 items in each study group (Table 2). Of those, 11 were common to both groups. The overlap increased as we moved toward the more favored items. The top 10 of the two groups featured eight common items and there was perfect overlap in the top six priorities, although their priority order differed slightly between groups. Each group’s seventh priority was absent from the other group’s subset. Agreement between the aggregate rank ordering of items in each group was moderately high (Krippendorff’s alpha = 0.811, 95% CI = 0.669–0.920), indicating similar item prioritization despite being derived from different assessment modes. All results of the third round on which the priority order in each group was based are provided in Additional file 2.

**Table 2:** Prioritization of top items in the study groups

	**Priority**	
	Rating group (*n* = 17)	Ranking group (*n* = 13)
{PPR1} Feeling that healthcare professionals are truly listening in order to tailor care according to the motivation and requests of each patient	1	1
{PT1} Healthcare professionals having up-to-date cardiovascular health training in their respective fields	2	4
{PPR3} Ensuring consistency in the professionals who follow the patient (same doctor, same nurse, etc.)	5	3
{SMS2} Receiving personalized information on your own cardiovascular health (personal check-up, origin and nature of the problem, risks, etc.)	6	2
{A2} Being able to reach a healthcare professional within 24–48 h in the event of a problem, either on site, by phone, videoconference or email	4	5
{IS4} Having a single, common medical record between all healthcare providers	3	6
{PC2} Ensuring effective collaboration between the clinic and pharmacists in the community	7	Not in subset
{SMS4} Receiving practical help to initiate lifestyle changes (nutritional evaluation, health literacy education service, etc.)	Not in subset	7
{A1} Being able to get an appointment with your family doctor on short notice	8	8
{PC1} Ensuring effective collaboration between family doctors and nurses at the clinic	8	10
{SN1} Obtaining short delays for examinations and consultations that must be done outside the clinic	Not in subset	9
{PC4} Ensuring effective collaboration between the clinic and specialist physicians (e.g., cardiologists)	12	10
{SN4} Coordinating the appointments (in and out of the clinic) to minimize the inconvenience to patients	10	13
{SMS3} Receiving training and tools to help you manage your own health (how to take your blood pressure, what to do based on your results, etc.)	11	Not in subset
{SN3} Having access to a variety of tests (blood tests, echocardiography, etc.) at the clinic without having to be referred externally	13	Not in subset
{PC3} Ensuring effective collaboration between family doctors and allied healthcare professionals specializing in healthy lifestyles	15	12
{SN5} Explaining the role of each healthcare professional and when/how to refer to the right person	14	Not in subset
{CTC4} Having a specialist in weight and obesity management available on the clinical team	Not in subset	14
{A4} Having access to all clinic services in the evening and on weekends	Not in subset	14
{CTC6} Having a nurse specialized in cardiovascular health available on the clinical team	16	Not in subset
{CTC2} Having a nutrition specialist available on the clinical team	Not in subset	16

Abbreviations in braces refer to item themes (*PPR* Patient-professional relationship, *PT* Professional training, *SMS* Self-management support, *A* Accessibility, *IS* Information systems, *PC* Professional collaboration, *SN* Services network, *CTC* Clinical team composition), and numbers to their sequential order within the theme

### Secondary outcomes

Table 3 summarizes results for secondary outcomes. The second-round questionnaire took on average about 20 min to complete in both groups, with no statistically significant difference between them (Mann–Whitney *U* = 121.0; *p* = 0.630). However, the mean time to complete the third-round questionnaire dropped to 12 min in the rating group but not in the ranking group, and this difference trended close to statistical significance (Mann–Whitney *U* = 64.0; *p* = 0.053). Rating panelists also reported the assessment task to be relatively easy, all scoring either 5 or 6 on the SEQ scale. SEQ scores of ranking panelists were more spread and indicated that they found the assessment task comparatively more difficult, a difference which was highly statistically significant (Mann–Whitney *U* = 17.5; *p* < 0.001).

**Table 3:** Differences in secondary outcomes

	**Second round**			**Third round**		
	Rating (*n* = 18)	Ranking (*n* = 15)	p*	Rating (*n* = 17)	Ranking (*n* = 13)	p*
**Questionnaire completion time, in minutes**						
Mean	19.5	22.2	0.630	12.1	21.0	0.053
SD	12.0	15.5		10.6	17.6	
Range	3.5–39.8	7.0–58.0		3.4–43.1	4.9–56.8	
**SEQ score**						
Median				6	4	0.00001
IQR				5–6	3–4.5	
Range				5–6	2–6	

*SD* Standard deviation, *SEQ* Single-Ease Question, *IQR* Interquartile range

^*^Mann–Whitney *U* tests (two-tailed exact significance)

## Discussion

### Main findings

In this randomized trial, we found moderately high reliability between the prioritization of top items in a rating arm and a ranking arm at the end of a Delphi process (Krippendorff alpha = 0.811, 95% CI = 0.669–0.920). Krippendorff states that it is customary to require alpha ≥ 0.8 to ensure that the data under consideration are similarly interpretable by different coders and that ≥ 0.667 is the lower limit where tentative conclusions are acceptable [45]. However, this statistic only considers comparison pairs, i.e., items reassessed in both groups. The subsets of top items did not fully coincide in the two groups. Significant discrepancies were found as early as the seventh top item and were more frequent among items of moderate importance. Some of these differences could be an artifact of the procedures used to select the items to include in the subsets. Rating panelists could give low or high ratings to all items in a given thematic list, whereas ranking panelists were forced to highlight a top and a bottom item in each list regardless of their absolute importance. We can speculate that less discrepancies may have occurred if ranking panelists had been asked to rank the items within fewer lists during round 2 (e.g., 20 items grouped under two themes). However, the increased cognitive load could have affected response quality, possibly resulting in further discrepancies. This underscores that rating and ranking may not be completely interchangeable, especially when moving away from items that are clearly superior or inferior to others. In this, our main results are consistent with studies comparing rating and ranking in the field of value systems measurement [27–29, 33–35]. Our study extends previous findings to the context of medical and health services research. To our knowledge, this is also the first experimental study to compare the two main assessment procedures available to quantify opinions in the context of a Delphi survey.

Contrary to common practice in Delphi, we did not define a consensus threshold to compare the final outputs of our experimental groups. Rather, we viewed the prioritization of top items as providing a fairer comparison between rating and ranking. Had we done so, it is likely that the final set of items in the ranking group would have included fewer items than in the rating group, as ranking prohibits ties. This is apparent in the results of the third round (Additional file 2), where the proportions of 6–7 ratings obtained for top items show a ceiling effect, whereas those of top-half rankings do not. Additionally, by focusing on item prioritization, we avoided undesirable interferences on the results that could have been caused by the number of response categories in the rating scale and the arbitrary choice of a cutoff point, as demonstrated in other methodological investigations of the Delphi technique [21, 22].

This study also found that the ranking task was significantly more difficult. The 9-min mean difference to complete the third-round questionnaire in favor of the rating group failed to achieve statistical significance (*p* = 0.053). The variance of questionnaire completion time was much larger than we had originally anticipated. Due to the limited number of participants in our trial, we were unable to achieve sufficient statistical power to identify a significant and meaningful difference on this secondary outcome. Although we cannot ascertain whether increasing the sample size would have led to a significant effect, it is reasonable to assume that a more challenging task would require more time to complete. Ranking requires to consider multiple items at once to situate them in relation to one another, while rating does not. Research in survey methodology has shown that the cognitive burden associated with ranking increases with the number of items to rank and as the difference in importance between them decreases, requiring tougher choices [48]. This is exactly what happened during our final round, as ranking panelists were faced with a larger list of more important items to reassess. Although we were unable to substantiate this in our study, it is conceivable that task difficulty could have a negative impact on the response rate, potentially compromising the representation of opinions of the invited panel. The effect may be more prominent in Delphi surveys with a higher number of rounds. This is an issue which could be explored in future work. Greater task difficulty may also negatively affect data quality by fostering satisficing behaviors in respondents, i.e., taking cognitive shortcuts instead of providing optimal answers [49, 50]. Although we did not set out to assess data quality in this trial, we did observe that a higher proportion of panelists in the rating group (14/18; 78%) provided justifications for their assessments during round 2, compared to the ranking group (7/15; 47%). This arguably translated into a richer exchange of information between rating panelists. According to research [50], satisficing is also more likely when higher task difficulty is combined with lower respondent ability and motivation. Thus, our trial results may have implications for conducting Delphi surveys with panels that are heterogenous in terms of cognitive ability. With many organizations now focused on diversity, equity, and inclusion, further research in this area is warranted.

### Strengths and limitations

The main strength of this study was the stratified random allocation of panelists to the rating or ranking study groups. This ensured both groups would be heterogenous and balanced in terms of clinical and experiential expertise as well as other potential confounders. Our Delphi panels also reflected the importance of acknowledging patients as equal partners with professionals in health systems improvement, a notion that is increasingly recognized [37, 51]. Other strengths include the random presentation of items in the second-round questionnaires which prevented framing effects [19] and the use of robust standard measures to assess trial outcomes.

This study also had some limitations. Firstly, although our panel sizes were consistent with recommendations for Delphi studies incorporating the sharing of arguments [9, 15, 39], the small sample size of the study limited the capacity to detect statistically significant effects on questionnaire completion time. Although our findings indicated the presence of a potential difference, a larger study is necessary to ascertain whether this effect is genuine or not. However, larger Delphi panels can pose their own problems. They have been associated with lower response rates [20] and can become suboptimal in terms of logistical and analytical resources required, due to the high volume of material needed to be reviewed after each round [15, 38]. Many Delphi studies restrict the scope of feedback to statistical information in order to accommodate larger panels, but this may be counterproductive, as Rowe et al. [44] argued, as it correspondingly limits the scope for improvements in group opinion. Nevertheless, greater numbers would have been preferable for the purpose of this trial. Secondly, task difficulty was only measured during round 3. Including the SEQ in round 2 would have allowed for comparing rating and ranking in a context that facilitates ranking, with shorter lists of more differentiated items. However, we believe that the results from our third round extrapolate best to ranking Delphi studies, which should include only the most important items on a consolidated list in the ranking rounds, following a trimming step if necessary [11, 25]. Thirdly, most of the dropouts were in the ranking group. However, they occurred equally before and after panelists were exposed to their assessment procedure, and they did not lead to statistically significant imbalances in the composition of the study groups. Finally, the panelists were only allowed to submit a maximum of five items in the first round and might have generated more items had they been allowed to do so. This restriction limited the number of items to assess in round 2. However, this feature was designed in accordance with the objectives of the main study [36], in order to focus the panelists on the most important organizational aspects and to reduce their response burden in subsequent rounds. The Delphi technique is very flexible. Even though our study was conducted in a realistic setting, the generalizability of the findings from this single trial is limited by its specific features.

## Conclusions

In this randomized trial, the use of a rating or ranking procedure led to a modestly similar prioritization of items in a Delphi survey with 30 panelists. We found that both experimental groups identified the same highest priorities but also that discrepancies became increasingly frequent when moving away from the most favored items. The ranking task was perceived as significantly more difficult. Time to complete the study questionnaires showed no statistically significant difference. The embedded trial design was restricted by the requirements of the main Delphi study. Our study should be replicated with a larger number of participants and with variations in the ranking and rating procedures (e.g., ranking within larger lists and rating with smaller, larger or bipolar scales). When determining how to assess items in future Delphi surveys, investigators may favor rating unless they specifically seek to avoid potential ceiling effects.

### Supplementary Information

**Additional file 1.** Round 2 results (Quantitative results obtained in the rating and ranking experimental groups during Round 2, with items included in the final round’s subset).

**Additional file 2.** Round 3 results (Quantitative results obtained in the rating and ranking experimental groups during Round 3, by rank-ordered priority).

## Data Availability

The dataset generated and/or analyzed during the current study is available in the Figshare repository, https://doi.org/10.6084/m9.figshare.21648620.

## Abbreviations

IQR: Interquartile range
SD: Standard deviation
SEQ: Single Ease Question
