# Supplementary material for: Rating versus ranking in a Delphi survey: a randomized controlled trial
Source: Trials. 2023 Aug 18;24:543. doi: 10.1186/s13063-023-07442-6 (PMC10436639; doi:10.1186/s13063-023-07442-6)
Supplement: Supplementary file 1 — Additional file 1. Round 2 results (Quantitative results obtained in the rating and ranking experimental groups during Round 2, with items included in the final round’s subset). [file 13063_2023_7442_MOESM1_ESM.docx]

**Proportions of ratings of 6 or 7 obtained for the items during Round 2 in the rating group (n = 18 panelists), in descending order.**

| **Organizational items** | **% 6-7 ratings** | **Kept in Round 3 subset?** |
| --- | --- | --- |
| {PPR1} Feeling that healthcare professionals are truly listening in order to tailor care according to the motivation and requests of each patient | 94.1 | YES |
| {IS4} Having a single, common medical record between all healthcare providers | 94.1 | YES |
| {PT1} Healthcare professionals having up-to-date cardiovascular health training in their respective fields | 88.3 | YES |
| {PC1} Ensuring effective collaboration between family doctors and nurses at the clinic | 83.3 | YES |
| {PC2} Ensuring effective collaboration between the clinic and pharmacists in the community | 83.3 | YES |
| {A1} Being able to get an appointment with your family doctor on short notice | 82.4 | YES |
| {A2} Being able to reach a healthcare professional within 24-48 hours in the event of a problem, either on site, by phone, videoconference or email | 82.4 | YES |
| {PPR3} Ensuring consistency in the professionals who follow the patient (same doctor, same nurse, etc.) | 82.3 | YES |
| {SN4} Coordinating the appointments (in and out of the clinic) to minimize the inconvenience to patients | 76.4 | YES |
| {SMS2} Receiving personalized information on your own cardiovascular health (personal check-up, origin and nature of the problem, risks, etc.) | 76.4 | YES |
| {CTC6} Having a nurse specialized in cardiovascular health available on the clinical team | 76.4 | YES |
| {SN5} Explaining the role of each healthcare professional and when/how to refer to the right person | 70.6 | YES |
| {SMS3} Receiving training and tools to help you manage your own health (how to take your blood pressure, what to do based on your results, etc.) | 70.5 | YES |
| {PC3} Ensuring effective collaboration between family doctors and allied healthcare professionals specializing in healthy lifestyles | 66.7 | YES |
| {PC4} Ensuring effective collaboration between the clinic and specialist physicians (e.g., cardiologists) | 66.7 | YES |
| {SN3} Having access to a variety of tests (blood tests, echocardiography, etc.) at the clinic without having to be referred externally | 64.7 | YES^*^ |
| {SMS4} Receiving practical help to initiate lifestyle changes (nutritional evaluation, health literacy education service, etc.) | 58.9 | NO |
| {CTC2} Having a nutrition specialist available on the clinical team | 58.9 | NO |
| {SN1} Obtaining short delays for examinations and consultations that must be done outside the clinic | 58.8 | NO |
| {PT3} Having a doctor with sufficient clinical experience in cardiovascular health | 58.8 | NO |
| {IS3} Being able to send and receive information electronically with the clinic (email, texting) regarding health status, test results, notifications for follow-ups, etc. | 58.8 | NO |
| {CFU4} Offering help in managing health-related stress and anxiety | 53.0 | NO |
| {SN2} Having access to inexpensive or free resources and programs to improve the health and lifestyle of people with a cardiovascular health condition | 52.9 | NO |
| {A6} Being seen on time for an appointment with little or no delay | 47.1 | NO |
| {A5} Having access to all clinic services in French or English | 47.0 | NO |
| {CFU1} Having protocols in place to systematically direct patients to the right care and services based on their condition | 47.0 | NO |
| {PC5} Ensuring effective collaboration between the clinic and community resources | 44.5 | NO |
| {A7} Free parking near the clinic | 41.2 | NO |
| {CTC1} Having a pharmacist available on the clinical team | 41.2 | NO |
| {CTC4} Having a specialist in weight and obesity management available on the clinical team | 41.2 | NO |
| {IS1} Easy access for patients to their medical records | 41.2 | NO |
| {IS2} Having a dedicated phone support line for registered patients where nurses would have access to the patients’ records | 41.2 | NO |
| {PPR2} Involving the patient’s family and loved ones in care | 41.1 | NO |
| {CTC3} Having a physical activity specialist available on the clinical team | 35.3 | NO |
| {CTC5} Having a smoking cessation specialist available on the clinical team | 35.3 | NO |
| {A4} Having access to all clinic services in the evening and on weekends | 35.2 | NO |
| {A3} Having the option to get longer consultations | 29.4 | NO |
| {CFU2} Conducting regular follow-ups on the progress made or not (e.g., in a logbook detailing steps of care) | 29.4 | NO |
| {CFU3} Offering activities at the clinic on healthy lifestyle and prevention of cardiovascular health problems | 29.4 | NO |
| {SMS1} Receiving general information on cardiovascular health and available support resources | 29.4 | NO |
| {PT2} Having a doctor who is better trained to provide counseling on nutrition and physical activity | 29.4 | NO |

^*^Item met the inclusion criteria (rated 6 or 7 by two-thirds of panelists) after accounting for missing data.

Abbreviations in braces refer to item themes (A: accessibility; CFU: Care and Follow-Up; CTC: clinical team composition; IS: information systems; PC: professional collaboration; PPR: patient-professional relationship; PT: professional training; SMS: self-management support; SN: services network), and numbers to their sequential order within the theme.

**Proportions of top-half rankings obtained for the items during Round 2 in the ranking group (n = 15 panelists), in descending order.**

| **Organizational items** | **% top-half rankings** | **Kept in Round 3 subset?** |
| --- | --- | --- |
| {PPR3} Ensuring consistency in the professionals who follow the patient (same doctor, same nurse, etc.) | 100.0 | YES |
| {A2} Being able to reach a healthcare professional within 24-48 hours in the event of a problem, either on site, by phone, videoconference or email | 93.3 | YES |
| {SMS2} Receiving personalized information on your own cardiovascular health (personal check-up, origin and nature of the problem, risks, etc.) | 86.7 | YES |
| {PC1} Ensuring effective collaboration between family doctors and nurses at the clinic | 86.7 | YES |
| {PT1} Healthcare professionals having up-to-date cardiovascular health training in their respective fields | 86.7 | YES |
| {PPR1} Feeling that healthcare professionals are truly listening in order to tailor care according to the motivation and requests of each patient | 86.7 | YES |
| {A1} Being able to get an appointment with your family doctor on short notice | 80.0 | YES |
| {SN1} Obtaining short delays for examinations and consultations that must be done outside the clinic | 80.0 | YES |
| {SN4} Coordinating the appointments (in and out of the clinic) to minimize the inconvenience to patients | 80.0 | YES |
| {CTC2} Having a nutrition specialist available on the clinical team | 80.0 | YES |
| {PC4} Ensuring effective collaboration between the clinic and specialist physicians (e.g., cardiologists) | 80.0 | YES |
| {A4} Having access to all clinic services in the evening and on weekends | 73.3 | YES |
| {SMS4} Receiving practical help to initiate lifestyle changes (nutritional evaluation, health literacy education service, etc.) | 66.7 | YES |
| {CTC4} Having a specialist in weight and obesity management available on the clinical team | 66.7 | YES |
| {PC3} Ensuring effective collaboration between family doctors and allied healthcare professionals specializing in healthy lifestyles | 66.7 | YES |
| {IS4} Having a single, common medical record between all healthcare providers | 66.7 | YES |
| {SN3} Having access to a variety of tests (blood tests, echocardiography, etc.) at the clinic without having to be referred externally | 60.0 | NO |
| {CFU1} Having protocols in place to systematically direct patients to the right care and services based on their condition | 60.0 | NO |
| {PT2} Having a doctor who is better trained to provide counseling on nutrition and physical activity | 60.0 | NO |
| {IS2} Having a dedicated phone support line for registered patients where nurses would have access to the patients’ records | 60.0 | NO |
| {A6} Being seen on time for an appointment with little or no delay | 53.3 | NO |
| {SN2} Having access to inexpensive or free resources and programs to improve the health and lifestyle of people with a cardiovascular health condition | 53.3 | NO |
| {CTC6} Having a nurse specialized in cardiovascular health available on the clinical team | 53.3 | NO |
| {PT3} Having a doctor with sufficient clinical experience in cardiovascular health | 53.3 | NO |
| {IS3} Being able to send and receive information electronically with the clinic (email, texting) regarding health status, test results, notifications for follow-ups, etc. | 53.3 | NO |
| {CFU2} Conducting regular follow-ups on the progress made or not (e.g., in a logbook detailing steps of care) | 46.7 | NO |
| {CFU3} Offering activities at the clinic on healthy lifestyle and prevention of cardiovascular health problems | 46.7 | NO |
| {CFU4} Offering help in managing health-related stress and anxiety | 46.7 | NO |
| {SMS3} Receiving training and tools to help you manage your own health (how to take your blood pressure, what to do based on your results, etc.) | 46.7 | NO |
| {A5} Having access to all clinic services in French or English | 40.0 | NO |
| {CTC1} Having a pharmacist available on the clinical team | 40.0 | NO |
| {PC2} Ensuring effective collaboration between the clinic and pharmacists in the community | 40.0 | NO |
| {A3} Having the option to get longer consultations | 33.3 | NO |
| {CTC3} Having a physical activity specialist available on the clinical team | 33.3 | NO |
| {A7} Free parking near the clinic | 26.7 | NO |
| {SN5} Explaining the role of each healthcare professional and when/how to refer to the right person | 26.7 | NO |
| {CTC5} Having a smoking cessation specialist available on the clinical team | 26.7 | NO |
| {PC5} Ensuring effective collaboration between the clinic and community resources | 26.7 | NO |
| {IS1} Easy access for patients to their medical records | 20.0 | NO |
| {PPR2} Involving the patient’s family and loved ones in care | 13.3 | NO |
| {SMS1} Receiving general information on cardiovascular health and available support resources | 0.0 | NO |

Abbreviations in braces refer to item themes (A: accessibility; CFU: Care and Follow-Up; CTC: clinical team composition; IS: information systems; PC: professional collaboration; PPR: patient-professional relationship; PT: professional training; SMS: self-management support; SN: services network), and numbers to their sequential order within the theme.
