# Supplementary material for: Rating versus ranking in a Delphi survey: a randomized controlled trial
Source: Trials. 2023 Aug 18;24:543. doi: 10.1186/s13063-023-07442-6 (PMC10436639; doi:10.1186/s13063-023-07442-6)
Supplement: Supplementary file 2 — Additional file 2. Round 3 results (Quantitative results obtained in the rating and ranking experimental groups during Round 3, by rank-ordered priority). [file 13063_2023_7442_MOESM2_ESM.docx]

**Rank-ordered priorities in the rating group (n = 17 panelists) after Round 3.**

| **Priority** | **Organizational items** | **% 6-7 ratings** | **% 7-only ratings** |
| --- | --- | --- | --- |
| 1 | {PPR1} Feeling that healthcare professionals are truly listening in order to tailor care according to the motivation and requests of each patient | 100.0 | 58.8 |
| 2 | {PT1} Healthcare professionals having up-to-date cardiovascular health training in their respective fields | 94.1 | 52.9 |
| 3 | {IS4} Having a single, common medical record between all healthcare providers | 94.1 | 47.1 |
| 4 | {A2} Being able to reach a healthcare professional within 24-48 hours in the event of a problem, either on site, by phone, videoconference or email | 94.1 | 41.2 |
| 5 | {PPR3} Ensuring consistency in the professionals who follow the patient (same doctor, same nurse, etc.) | 94.1 | 23.5 |
| 6 | {SMS2} Receiving personalized information on your own cardiovascular health (personal check-up, origin and nature of the problem, risks, etc.) | 88.2 | 23.5 |
| 7 | {PC2} Ensuring effective collaboration between the clinic and pharmacists in the community | 88.2 | 17.6 |
| 8 | {A1} Being able to get an appointment with your family doctor on short notice | 88.2 | 11.8 |
| 8 | {PC1} Ensuring effective collaboration between family doctors and nurses at the clinic | 88.2 | 11.8 |
| 10 | {SN4} Coordinating the appointments (in and out of the clinic) to minimize the inconvenience to patients | 82.4 | 11.8 |
| 11 | {SMS3} Receiving training and tools to help you manage your own health (how to take your blood pressure, what to do based on your results, etc.) | 76.5 | 11.8 |
| 12 | {PC4} Ensuring effective collaboration between the clinic and specialist physicians (e.g., cardiologists) | 70.6 | 29.4 |
| 13 | {SN3} Having access to a variety of tests (blood tests, echocardiography, etc.) at the clinic without having to be referred externally | 70.6 | 23.5 |
| 14 | {SN5} Explaining the role of each healthcare professional and when/how to refer to the right person | 70.6 | 5.9 |
| 15 | {PC3} Ensuring effective collaboration between family doctors and allied healthcare professionals specializing in healthy lifestyles | 64.7 | 17.6 |
| 16 | {CTC6} Having a nurse specialized in cardiovascular health available on the clinical team | 64.7 | 11.8 |

Abbreviations in braces refer to item themes (A: accessibility; CTC: clinical team composition; IS: information systems; PC: professional collaboration; PPR: patient-professional relationship; PT: professional training; SMS: self-management support; SN: services network), and numbers to their sequential order within the theme.

**Rank-ordered priorities in the ranking group (n = 13 panelists) after Round 3**

| **Priority** | **Organizational items** | **% top-half rankings** | **Median rank** |
| --- | --- | --- | --- |
| 1 | {PPR1} Feeling that healthcare professionals are truly listening in order to tailor care according to the motivation and requests of each patient | 100.0 | 1 |
| 2 | {SMS2} Receiving personalized information on your own cardiovascular health (personal check-up, origin and nature of the problem, risks, etc.) | 100.0 | 3 |
| 3 | {PPR3} Ensuring consistency in the professionals who follow the patient (same doctor, same nurse, etc.) | 69.2 | 5 |
| 4 | {PT1} Healthcare professionals having up-to-date cardiovascular health training in their respective fields | 69.2 | 7 |
| 5 | {A2} Being able to reach a healthcare professional within 24-48 hours in the event of a problem, either on site, by phone, videoconference or email | 61.5 | 6 |
| 6 | {IS4} Having a single, common medical record between all healthcare providers | 61.5 | 8 |
| 7 | {SMS4} Receiving practical help to initiate lifestyle changes (nutritional evaluation, health literacy education service, etc.) | 53.8 | 6 |
| 8 | {A1} Being able to get an appointment with your family doctor on short notice | 53.8 | 8 |
| 9 | {SN1} Obtaining short delays for examinations and consultations that must be done outside the clinic | 46.2 | 9 |
| 10 | {PC4} Ensuring effective collaboration between the clinic and specialist physicians (e.g., cardiologists) | 38.5 | 10 |
| 10 | {PC1} Ensuring effective collaboration between family doctors and nurses at the clinic | 38.5 | 10 |
| 12 | {PC3} Ensuring effective collaboration between family doctors and allied healthcare professionals specializing in healthy lifestyles | 30.8 | 12 |
| 13 | {SN4} Coordinating the appointments (in and out of the clinic) to minimize the inconvenience to patients | 23.1 | 13 |
| 14 | {A4} Having access to all clinic services in the evening and on weekends | 23.1 | 14 |
| 14 | {CTC4} Having a specialist in weight and obesity management available on the clinical team | 23.1 | 14 |
| 16 | {CTC2} Having a nutrition specialist available on the clinical team | 7.7 | 14 |

Abbreviations in braces refer to item themes (A: accessibility; CTC: clinical team composition; IS: information systems; PC: professional collaboration; PPR: patient-professional relationship; PT: professional training; SMS: self-management support; SN: services network), and numbers to their sequential order within the theme.
